# Electronically Monitored Labial Dabbing and Stylet ‘Probing’ Behaviors of Brown Marmorated Stink Bug, *Halyomorpha halys*, in Simulated Environments

**DOI:** 10.1371/journal.pone.0113514

**Published:** 2014-12-04

**Authors:** Nik G. Wiman, Vaughn M. Walton, Peter W. Shearer, Silvia I. Rondon

**Affiliations:** 1 Department of Horticulture, Oregon State University, Corvallis, Oregon, United States of America; 2 Mid-Columbia Agricultural Research and Extension Center, Oregon State University, Hood River, Oregon, United States of America; 3 Hermiston Agricultural Research and Extension Center, Oregon State University, Hermiston, Oregon, United States of America; United States Department of Agriculture, Beltsville Agricultural Research Center, United States of America

## Abstract

Brown marmorated stink bug, *Halyomorpha halys* (Stål), (Hemiptera: Pentatomidae) is an invasive polyphagous agricultural and urban nuisance pest of Asian origin that is becoming widespread in North America and Europe. Despite the economic importance of pentatomid pests worldwide, their feeding behavior is poorly understood. Electronically monitored insect feeding (EMIF) technology is a useful tool in studies of feeding behavior of Hemiptera. Here we examined *H. halys* feeding behavior using an EMIF system designed for high throughput studies in environmental chambers. Our objectives were to quantify feeding activity by monitoring proboscis contacts with green beans, including labial dabbing and stylet penetration of the beans, which we collectively define as ‘probes’. We examined frequency and duration of ‘probes’ in field-collected *H. halys* over 48 hours and we determined how environmental conditions could affect diel and seasonal periodicity of ‘probing’ activity. We found differences in ‘probing’ activity between months when the assays were conducted. These differences in activity may have reflected different environmental conditions, and they also coincide with what is known about the phenology of *H. halys*. While a substantial number of ‘probes’ occurred during scotophase, including some of the longest mean ‘probe’ durations, activity was either lower or similar to ‘probing’ activity levels during photophase on average. We found that temperature had a significant impact on *H. halys* ‘probing’ behavior and may influence periodicity of activity. Our data suggest that the minimal temperature at which ‘probing’ of *H. halys* occurs is between 3.5 and 6.1°C (95% CI), and that ‘probing’ does not occur at temperatures above 26.5 to 29.6°C (95% CI). We estimated that the optimal temperature for ‘probing’ is between 16 and 17°C.

## Introduction

Phytophagous stink bugs (Hemiptera: Pentatomidae) can be highly destructive pests on a wide range of crops worldwide [1]. Their feeding can cause many different types of crop damage, and is particularly destructive on the fruiting parts of the plant [2]. Each time the stylets are inserted into plant tissues, a puncture will result, and secondary infection from incidental or vectored yeasts can result in tissue decay or necrosis [3], [4]. Pentatomidae can vector trypanosomatid flagellate pathogens as well as phytoplasms and fungi that can cause disease in host plants [5]. Salivary secretions, particularly enzymatic watery saliva, dissolve plant tissues and cause subsurface corking damage on fruits, vegetables, and nuts [11]. The saliva can also be considered phytoaggressive in plants and it can enzymatically disrupt function of plant tissues [6]–[8]. Feeding by stink bugs can also cause other deformities such as discoloration, or dimpling and “catfacing” on the surface of the fruits such as peaches [9]. Typically this damage occurs when undamaged fruit tissue continues to grow and expand around damaged tissue at a feeding site. In cotton, feeding by the stink bug *Nezara viridula* (L.) can cause blister damage [10]. Stink bug feeding can also cause tillering or stunting of developing corn and soybean plants, which negatively affects yield [11]. Feeding during the early stages of fruit or nut development can cause shriveling or abortion [2], [12]. Finally, maturation of crops can be affected by stink bug feeding damage, with soy showing delayed maturity [13], and tomato showing premature maturation [14].

Brown marmorated stink bug, *Halyomorpha halys* (Stål), (Hemiptera: Pentatomidae) is an invasive polyphagous agricultural and urban nuisance pest of Asian origin [15], [16]. Many regions of the world are environmentally suitable for this insect, and this pest has potential to severely damage a wide range of crops [17]. Nuisance problems occur when the insects aggregate en masse in structures for diapause, or are destructive on ornamental hosts or gardens [17]–[19]. The current range of this pest includes parts of Europe, Canada, and most US states [20], [21]. To date, the most significant economic effects in the US have been in tree fruit crops such as apples and peaches in Mid-Atlantic States [20]. *Halyomorpha halys* has been known from Oregon since 2004, and it now occurs in commercial tree crops, small fruits, vegetables, and generates many homeowner complaints [22]. The economic impact of *H. halys* is expected to increase dramatically as populations continue to spread and increase in other specialty crop production regions in the Western US and the world.

With the increasing economic importance of Pentatomoidea as a result of the spread the invasive pests *H. halys* [20], [21], *N. viridula* [23], *Megacopta cribraria* (Fabricius) [24], [25], and *Bagrada hilaris* (Burmeister) [26]–[29], research on feeding behavior of these insects is critical. Currently, much of what is known with regards to periodicity, frequency and duration of feeding activity for these insects has been determined by intermittent or constant visual observation of feeding over 24 hour periods [30]–[34]. However, Shearer and Jones [35] demonstrated that electronically-monitored feeding can be used to quantify periodicity of select feeding behaviors in *N. viridula* very effectively [35]. Electronically monitored insect feeding behavior (EMIF) originated as a methodology to study feeding in piercing-sucking insects [36]. In EMIF, the insect is placed into an electrical circuit that is completed only when the proboscis contacts the host plant. This provides data on the timing, duration and frequency of select feeding behaviors of these insects. In its most basic application, EMIF is similar to basic visual observations of feeding behavior as it records when the stylets are in contact with the food item and does not discriminate different feeding behaviors [35]. More precision can be obtained with electrical penetration graph (EPG) monitors, the dominant modern form of EMIF. EPG can measure changes in resistance and biopotential in the circuit that is completed by an insect feeding on an electrified host plant [37]. EPG produces high resolution data containing waveforms that can be precisely correlated with specific feeding behaviors such as penetration, salivation, fluid uptake, ingestion, and can yield important information about pathogen transmission [36], [38], [39].

EPG has primarily been a tool used for Hemiptera from suborders Sternorrhyncha and Auchenorrhyncha (previously Homoptera), such as aphids (Aphididae), planthoppers (Fulgoroidea), leafhoppers (Membracoidea), whiteflies (Aleyrodidae) and psylla (Psyllidae) [37]. Excluding leafhoppers, these insects ted to be true salivary sheath feeders, where the stylets are inserted into phloem or xylem without rupturing intermediate cells and a complete salivary sheath is formed [40]–[42]. Pentatomoidea are very versatile feeders and may use different feeding strategies depending on the plant tissue targeted [43], [5], [44]. To feed on vascular tissue, the strategy resembles salivary sheath feeding, but the stylets or salivary osmotic potential may be used to rupture mesophyllic or phloem cells. When feeding on tissue such as endosperm in seeds, there is greater reliance on “lacerate-and-flush” strategy where mechanical and enzymatic breakdown of cells is used and salivary sheaths are incomplete. *Halyomorpha halys* has been observed to feed on leaves, stems, petioles, even through bark [45]. However, pentatomid feeding on fruits, nuts, pods, and seeds, tends to cause the greatest economic crop damage [1], [2], [46]. Thus, reproductive plant structures are a focal site for the plant/insect interaction for pentatomids, and EMIF systems that can work on fruits and vegetables are important to improve knowledge of their feeding behavior.

In this study, we examined the feeding activity of field-collected *H. halys* using an EMIF system that was designed to continuously monitor feeding behavior of Pentatomidae on cut fruits and vegetables with minimal handling of insects. Our system has high replication potential and can function in environmental chambers. The feeding behaviors we monitored were limited to stylet contact with the food and stylet penetration with the food. Hereafter, we use the word ‘probe’ and derivative forms to include all published definitions of the word, *i.e.,* exploratory or sensory stylet contacts or labial dabbing, and stylet penetration of the food [37]. Our objectives were to 1) describe ‘probing’ activity patterns of *H. halys* in terms of frequency, duration, and total time spent ‘probing’ per day, 2) to examine how environmental conditions could affect ‘probing’ activity, and 3) examine how changes in environmental conditions affect diel periodicity of ‘probing’ activity over a season. These data are intended to improve our understanding of feeding behavior for *H. halys*. This information will help optimize sampling and management, and will provide a foundation for further studies on how feeding behavior relates to crop damage from this pest.

## Materials and Methods

The design of our EMIF activity monitor was modified from Shearer and Jones [35], with some refinements in configuration and instrumentation. A common concept between the aforementioned study, earlier studies [47]–[49], and the current study, is that the insect is confined to forage on a conductive metal screen attached to a power source. On the other side of the screen, a food item connected by wire to a recording instrument is placed within reach of the insect’s proboscis, but not close enough to touch the screen. When the insect commences ‘probing’ of the food item, the circuit becomes closed. When the circuit is closed, current flows from the power source, into the screen, through the tarsi and mouthparts of the insect, into the food item and the wire until the current is finally received and logged at the recording device. Conveyance of substrate current to the insect is reversed from typical EPG applications where the power source is connected to the host plant.

### Electronic feeding tables

**Figure 1:**
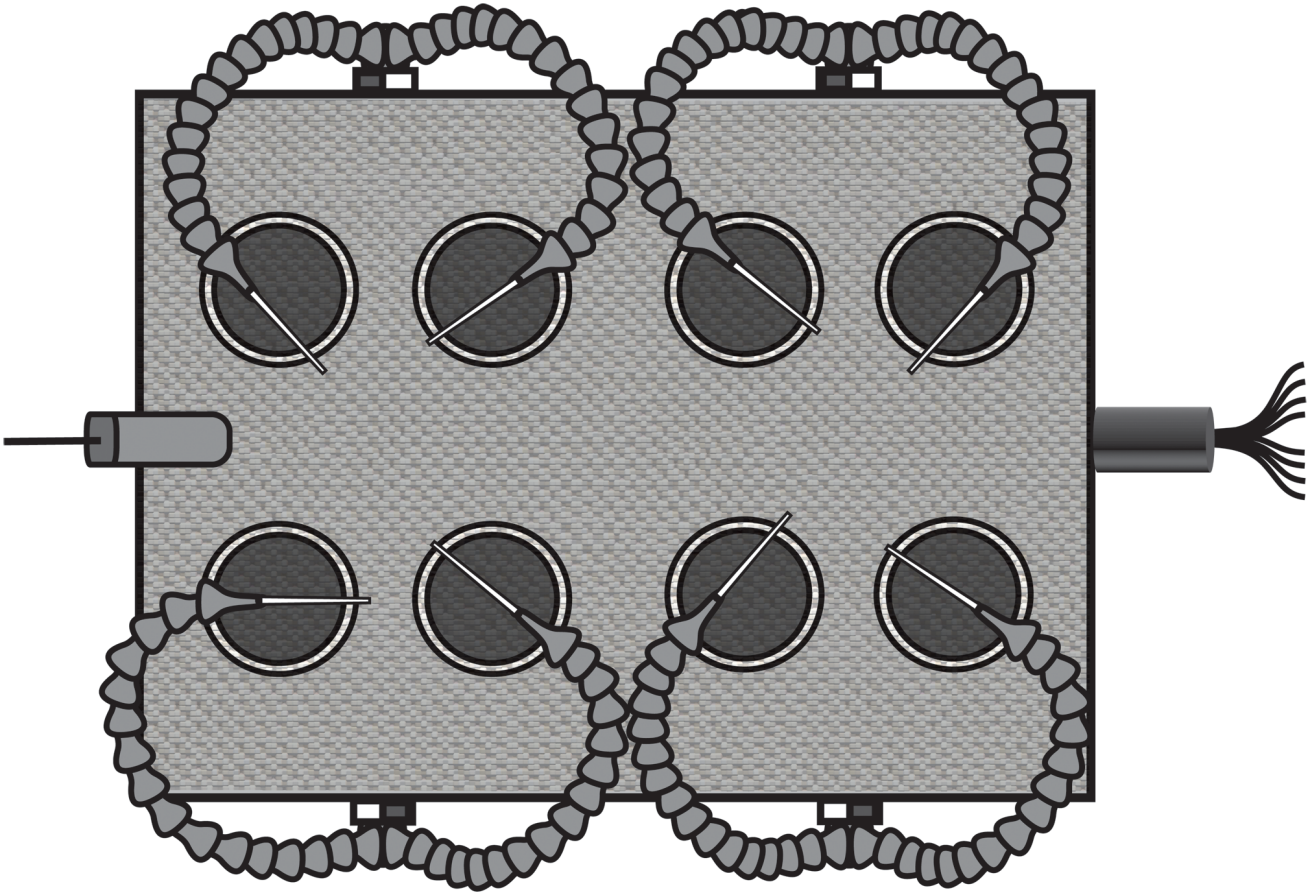
Top view of a feeding table.

**Figure 2:**
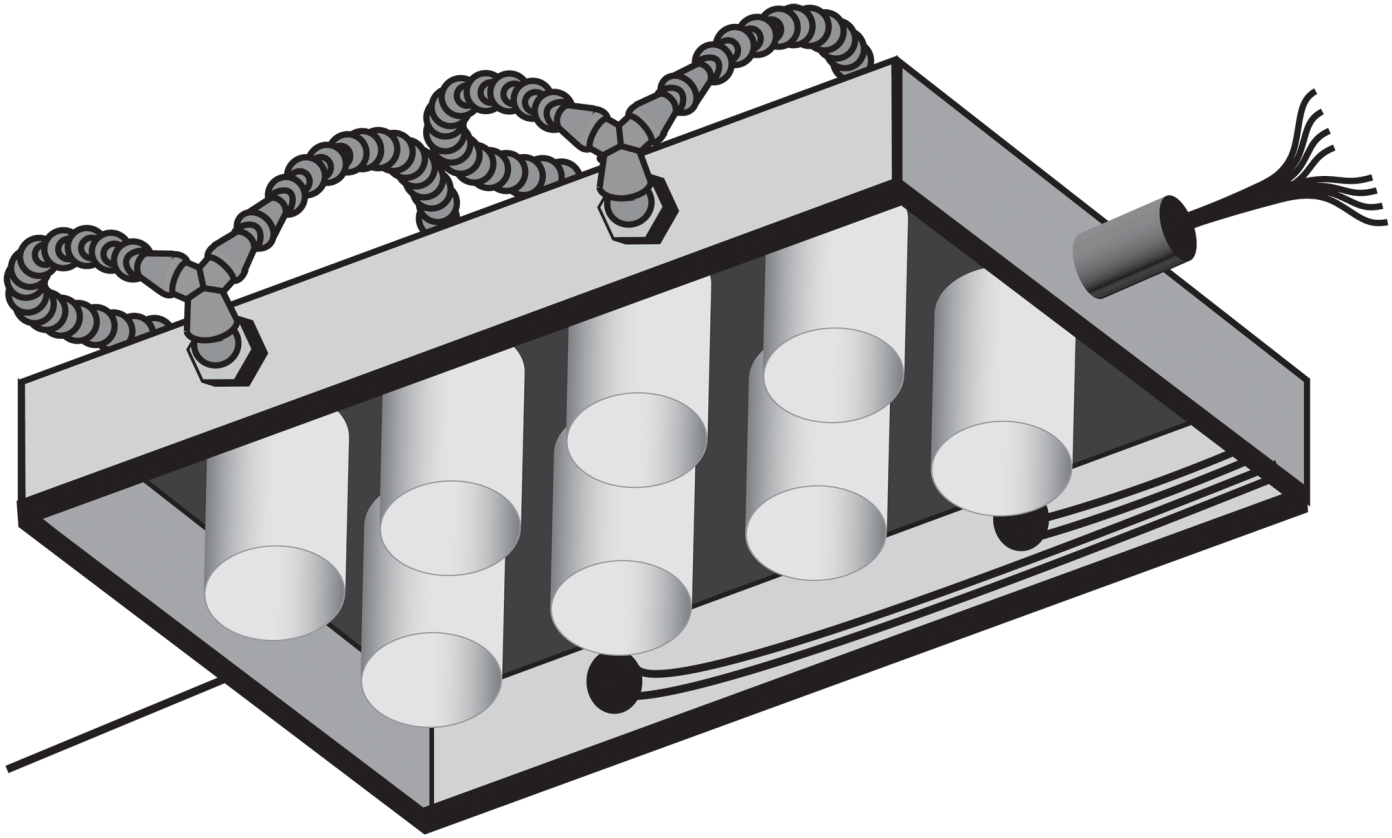
Bottom view of a feeding table.

Configuration of our EMIF system differs from systems used in previous studies in that we used two self-contained ‘feeding tables’, each with eight individual feeding ‘stations’. Feeding tables were essentially boxes that allowed compact layout of feeding stations so that feeding activity of eight individual insects per table could be monitored concurrently in environment chambers. Two feeding tables were constructed of wooden boxes (38.75 cm length, 25.5 cm width) and each contained eight holes (57 mm diameter), which accommodated tightly fitting screw caps lids (S-14506 ULINE, Pleasant Prairie, WI) and white polypropylene jars (120 ml; Figure 1). The centers of the fitted cup lids contained a smaller 50 mm diameter hole, leaving a threaded ring in the wood hole allowing the cups to be easily screwed into place from under the box to place *H. halys* into the feeding station (Figure 2). The entire top of each box was covered with a sheet of woven copper mesh (0.43 mm strand, 1 mm opening) that was stapled tightly to the box (Figure 1). Two portholes (½ in, 127 mm) were drilled into the long side of each box at the mid point between the first and second pair of holes to accommodate the electrode assemblies. The electrode assemblies consisted of two chains of ¼ in (64 mm) Loc-Line (LP-41401, Lockwood Products, Lake Oswego, OR) articulated plastic tubing segments, and these were joined by a Y fitting (LP-41408) and threaded into a port with a coupler (LP-41406). The tip of each electrode assembly was fitted with a nozzle (Loc-Line, LP-41403) and a 7.62 cm segment of solid copper wire (10 Ga, Artistic Wire, Coatesville, PA) soldered to a length of braided electrical wire (16 Ga.) was threaded through the port in the feeding table and through each tube segment and nozzle until just the large-gauge copper wire was protruding from the nozzle. A drop of hot glue applied between this wire electrode and the nozzle tip prevented the wire from sliding into the nozzle. A central port (190.5 mm) was added to one of the short sides of each box (“front”), and through this opening all of the wires from the individual electrodes were routed (Figure 1, 2). An automotive 9-pin male/female quick-connect coupler (with one wire removed) was attached to the 8 electrode lead wires, with a unique color representing each electrode and station. The other end of the coupler was wired to a 3 m long bundle of 8 wires representing the different electrodes. Wire bundles were wrapped with electrical tape and cable sleeves. The wire bundles connected to each box (8 wires each) were wired directly to the analog input (AI) terminals of a single USB data acquisition instrument (USB-6210, National Instruments Corporation, Austin, TX), which was connected to a PC via USB port. A regulated DC power supply (Mastech HY3003D, Acifica, Inc., San Jose, CA) was set to deliver +5 VDC to a steel L bracket that was screwed into table surface to serve as a power connection to the copper screen through a wire (16 Ga) and alligator clip. This single common screen served as the sole power substrate for each station on each table.

**Figure 3:**
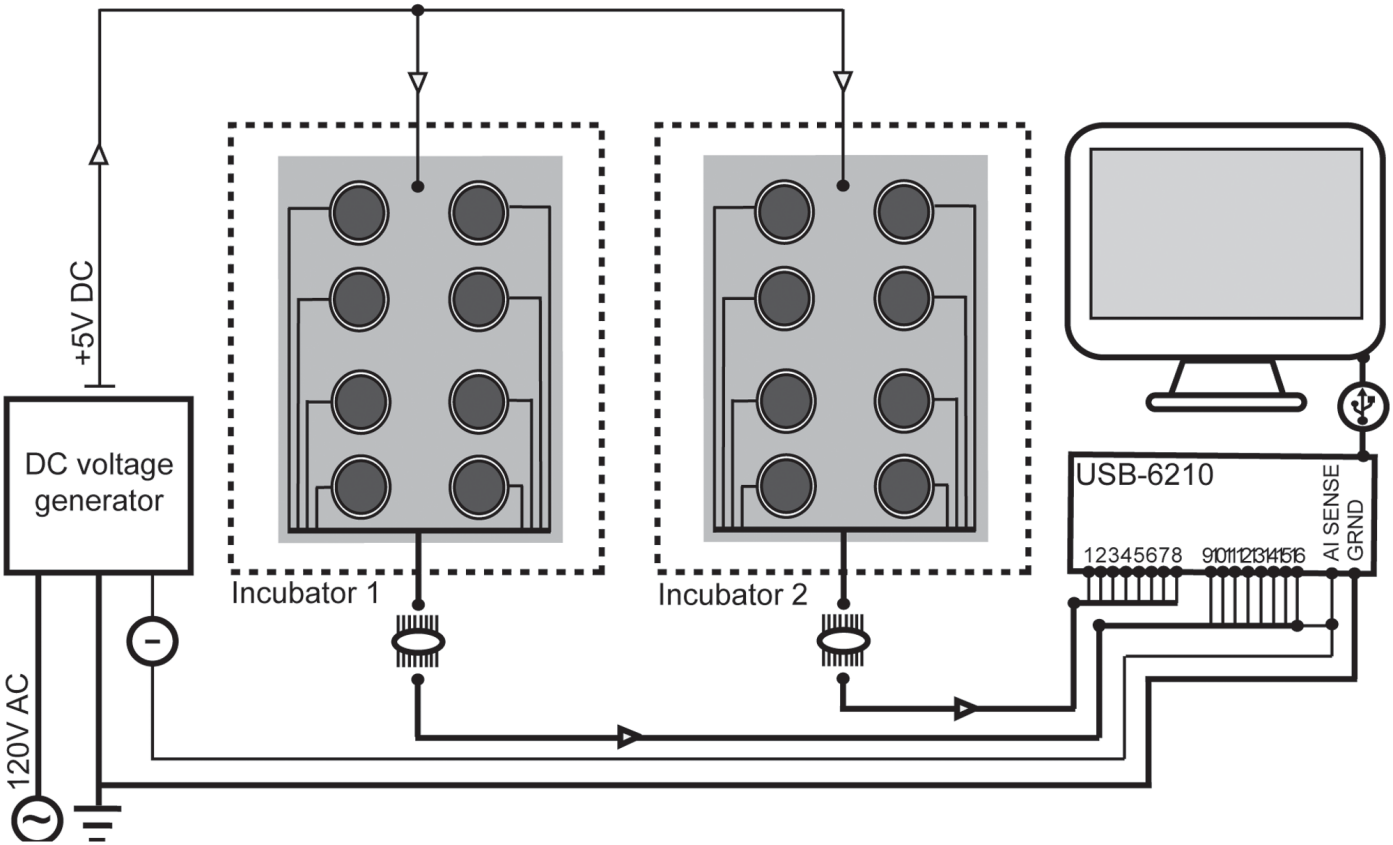
Wiring diagram for the EMIF system including hardware.

The ground terminal on the DC power supply was wired to the ground terminal on the USB-6210. The AI SENSE terminal on the USB-6210 was wired to one of the analog channels and to the black (−) terminal on the power supply to create a non-referenced single-ended (NRSE) input configuration ([50]; Figure 3). In this configuration there were 16 feeding stations on the two feeding tables. However, there were only 15 functional feeding stations as one channel on the USB-6210 was sacrificed to connect with the AI SENSE terminal, which reduced ambient signal noise. The resistance of the circuit elements between the power source and the copper screen, and between the electrode and the USB-6210 were approximately 0.5 Ω. The green beans that were attached to the electrodes as the food source provided a mean resistance of 12.36×10^6^ Ω (±0.57 SEM) between the electrode and surface of the bean. Analog input impedance on the USB-6210 is greater than 10×10^9^ Ω per channel [51], so the total input resistance (R_i_) in each circuit was more than 10.012×10^9^ Ω (excluding resistance added by the feeding insect). Resistance is high enough between the surface of tarsi and mouthparts in *H. halys* anesthetized by CO_2_ that it cannot be measured with a multimeter (max = 200×10^6^ Ω; Aide Tek VC97, Syncont. Inc., Parlin NJ). Thus, when the insect touched the surface of the bean with its mouthparts, it would experience a current of less than 4.99×10^−10^ A, or 0.0005 µA (A = V/Ω; Ohm’s law). This current is equivalent to using 500 mV on a standard commercial 1×10^9^ Ω DC-EPG system (5×10^−10^ A). The resistors represented by the insect and the bean on both sides of the gap in the circuit between the screen and the food precluded the possibility that false readings could occur. If the bare electrode (no bean) were placed within one millimeter of the screen, only 2–4 mV could be detected from the air in the space, thus static electricity would not generate signals above what would have been classified as noise in the analysis (see data analysis below).

### Data acquisition

A data acquisition program was written in the LabVIEW 2012 Version 12.01 (National Instruments Corp.) development environment. Because of delayed discharge between channels, rapid sampling between terminals on the USB-6210 device (250 kS/s scan rate), and high impedance in the circuits, signal response on neighboring channels (“crosstalk”) was initially a problem. We addressed this by modifying the driver configuration for the USB-6210 by adding dummy channels to increase inter-channel sampling delay. This allowed the multiplexer in the USB-6210 to fully discharge (“settle”) between readings so that the signals on the individual channels would not interfere with each other. For the purposes of this experiment, we acquired voltage readings at one-second intervals along with a date and time stamp. All data were output into comma-separated-value format. A front panel for the data acquisition program was designed to allow the user to set the duration of the feeding trial, and to set a threshold voltage to indicate feeding. Two line graphs showed voltage readings from the electrode in real-time for each station (8 stations each) and allowed the user to determine the stations that displayed feeding activity. Additionally, LED lights, one per station, were triggered when the insect was feeding. The LED’s illuminated when channel voltage exceeded the threshold set by the user.

### Food source

Fresh, organic green beans (*Phaseolus vulgaris* L.) were used as the food. Straight lengths of bean (approx. 7–10 cm) were cut from the stem, and the cut ends were wrapped tightly in Parafilm (Pechiney Plastic Packaging Company, Chicago IL). This kept beans fresh throughout each 48 h experimental period. Beans were pierced with a dissecting needle through the Parafilm in the center of the bean to pilot a straight hole for the electrode. A prepared bean was placed on each electrode and the assembly was positioned so that the maximum surface are of the bean was close to the copper screen without making contact with it (∼2 mm gap).

### Environmental chambers

Each feeding table was placed into a programmed environmental chamber (0.8 m^3^ interior vol.; CU-22, Percival Scientific, Inc., Perry IA). Monthly temperatures within each chamber represented a typically warm or cold average environment found in the Willamette Valley, OR relative to 30-year means (NOAA, Table 1). Environmental controls were adjusted every month for the duration of the study to most closely represent daily ambient conditions. There were nine daily temperature and three light intensity adjustments. Temperature steps were based on a sinusoidal representation of daily temperature cycles and were dependent on daily sunrise and sunset settings. Photoperiod timings were similarly adjusted on a monthly basis and were similar in the warm and cool environmental chambers (Table 1). Sunrise and sunset simulations were recreated by dawn and dusk light intensities (45 PPFD; photosynthetic photon flux) based on U.S. Naval Observatory Astronomical Applications Department projections for W123.16, N44.34 at 1 hr periods. Light intensity for the remainder of photophase was 85 PPFD. The coolest temperature was always represented at the last step before simulated sunrise and the warmest temperature was always represented at the step following the midpoint of photophase, thus simulating the thermal delay phenomenon. The other temperature steps represented gradual temperature increments between the highs and lows on the sine curve. Humidity was maintained at 70%.

**Table 1:** Environmental conditions in the warm and cool environmental chambers for each month of the experiment.

	Warm cab.			Cool cab.			Photoperiod			
Mo	High	Low	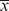	High	Low	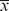	Photo.	Scoto.	L(h)	D(h)
May	22	11	17	16	7	12	05∶40	20∶30	14.9	9.1
June	26	13	20	23	9	16	05∶20	21∶00	15.6	8.4
July	31	15	23	26	11	19	05∶40	21∶00	15.3	8.7
Aug	28	13	21	23	9	16	06∶10	20∶20	14.2	9.8
Sept	25	11	18	18	7	13	06∶50	19∶20	12.5	11.5
Oct	17	7	12	14	3	9	07∶30	18∶20	10.9	13.1

### Collection of insects

*Halyomorpha halys* were collected with beating trays from host plants in the Willamette Valley [52] and maintained overnight at 4.5°C before placement in feeding tables the following morning. We did not consider the insects to be starved after this period of 15–16 hrs without food because of the lack of activity exhibited by *H. halys* at this holding temperature.

### Data analysis

A program was written in the open source statistical environment R [53] to retrieve all voltages and timestamps for sequences of voltages above the threshold of 1.5 V DC for each station over the 48 h assay from the CSV output files representing each trial. Observationally, this was the voltage threshold determined to correlate with ‘probing’ behavior. If voltages above the threshold were separated by more than 15 s, they were considered disparate ‘probing’ bouts. Voltage data from were merged with environmental data, which was collected with a HOBO data logger (UA-002-64, Onset Computer Corp., Bourne MA) using R package “xts” [54]. Analyzed variables were the number of ‘probes’, the duration of individual ‘probes’, and the total time spent ‘probing’. Statistical analyses on these variables were standard linear methods including ANOVA and Fisher’s LSD with log transformations to improve data normality where necessary. The number of *H. halys* that probed at each temperature across the range of temperatures tested over the course of this study was fit with a polynomial model to produce a temperature-dependent ‘probing’ response curve. The polynomial was solved to find the x intercepts that indicated the lower and upper feeding threshold, and the optimal ‘probing’ temperature was determined visually. We also looked at diel periodicity, where data were pooled into consecutive 2-hr increments to improve visualization of trends. To test diel periodicity of ‘probing’ activity, means of variables that were pooled within scotophase and within photophase were compared using Welch’s t-test, assuming that variances were unequal given more samples in the photophase data [35]. In one case, testing of the proportion of *H. halys* feeding during scotophase and photophase was accomplished using a binomial exact test in lieu of the t-test. Because photoperiod varied between the hours of 06∶00 and 07∶00, as well as 20∶00 and 21∶00 for the different months (Table 1), these transitional periods were excluded from the pooled data from scotophase and photophase and the t-test. All analyses were conducted using R.

## Results

### ‘Probing’ activity

**Figure 4:**
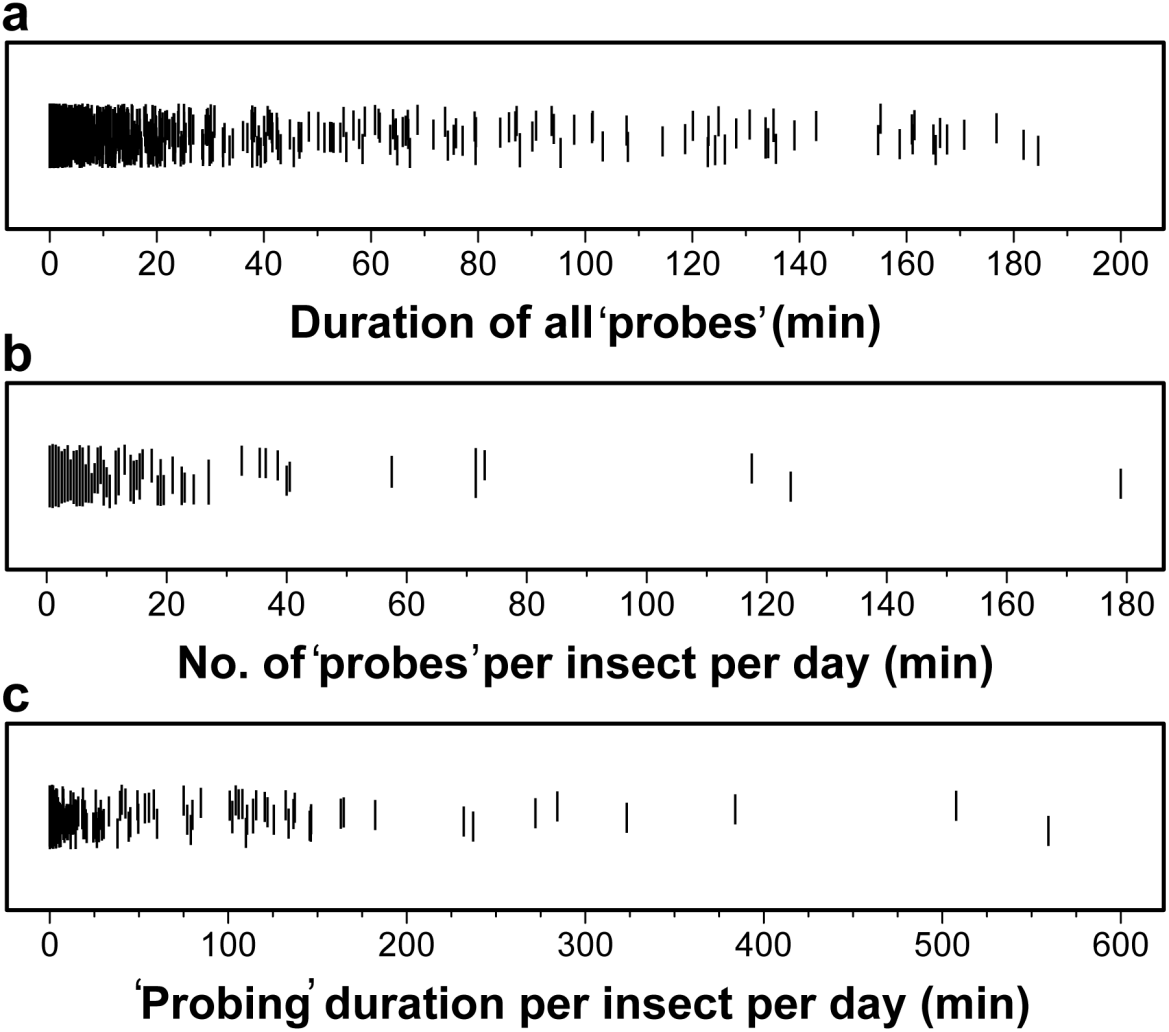
Length of all discrete ‘probes’ by *H. halys* (a), number of ‘probes’ per insect (b), and the total time spent ‘probing’ per day (c).

We defined ‘probing’ in the broadest sense such that we included any behavior where the mouthparts contacted the food [41]. Of all of the 302 field-collected adult *H. halys* placed in feeding tables in this experiment, 184 ‘probed’ at least once over the 48 h assay (61% response). Only responsive insects were included in the analyses. Individual adult *H. halys* ‘probe’ durations ranged from 1 sec to 184.6 min. The majority of the observed ‘probes’ were relatively short in duration; the arithmetic mean duration was 3.97 min (±0.51, 95% CI; Figure 4a). Individual *H. halys* ‘probed’ 1–179 times per 24 h in the 48 h assay. The arithmetic mean was 9.77 ‘probes’ every 24 h (±2.79, 95% CI; Figure 4b). Summing the total duration of ‘probes’ for individual insects indicated adults spent between 1 and 559.4 min (9.32 h) ‘probing’ per day, however the arithmetic mean duration was 38.8 min per 24 h (±12.03, 95% CI; Figure 4c). ‘Probing’ activity data was highly skewed toward zero, and log transformation was used to improve normality for subsequent significance testing on these variables. Skew of pooled duration data toward zero meant that back-transformed means (geometric) shown in plots were considerably lower than arithmetic means reported above.

### Environmental effects on ‘probing’ activity

**Figure 5:**
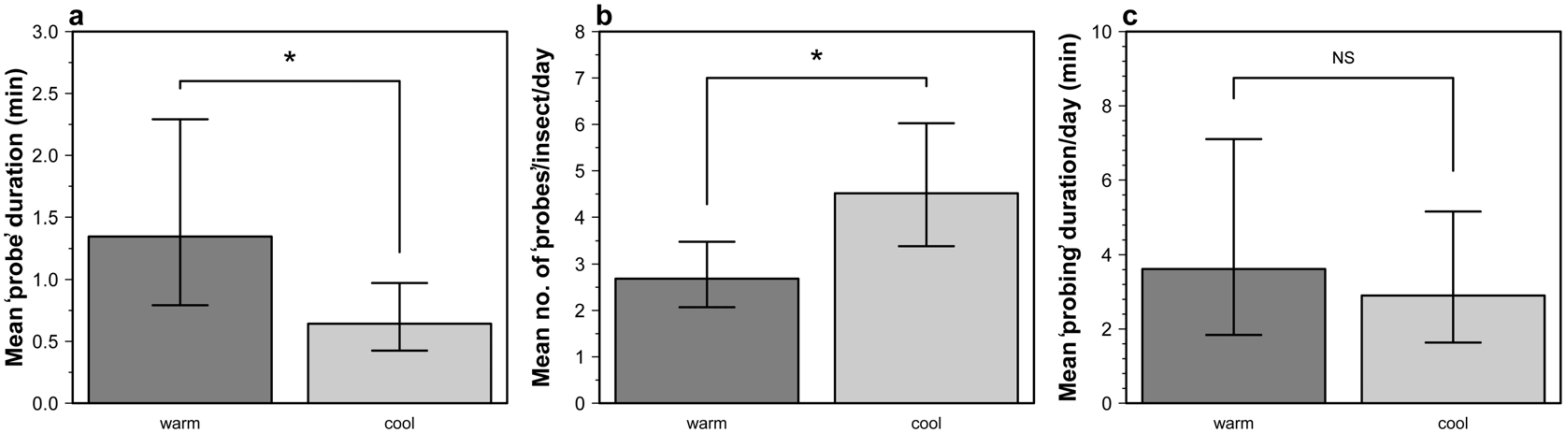
Means (±95% CI) for environmental chambers; ‘probing’ duration (a), ‘probing’ frequency (b), and sum ‘probing’ by *H. halys*. *P<0.05, NS = not significant (c).

There were significant differences in activity between insects depending on the temperature conditions in the environmental chambers (Table 1). *Halyomorpha halys* that were held in the warmer environmental chamber ‘probed’ for longer periods on average compared to insects held in the cooler chamber (*F* = 4.81; d.f. = 1,182; *P* = 0.029; Figure 5a). However, insects ‘probed’ more frequently in the cooler chamber (*F* = 6.43; d.f. = 1,182; *P* = 0.012; Figure 5b), so that overall, there was no difference in the mean ‘probing’ duration per insect per day (24 h) between the insects subject to the two environmental chambers (*F* = 0.232; d.f. = 1,182; *P* = 0.631; Figure 5c).

**Figure 6:**
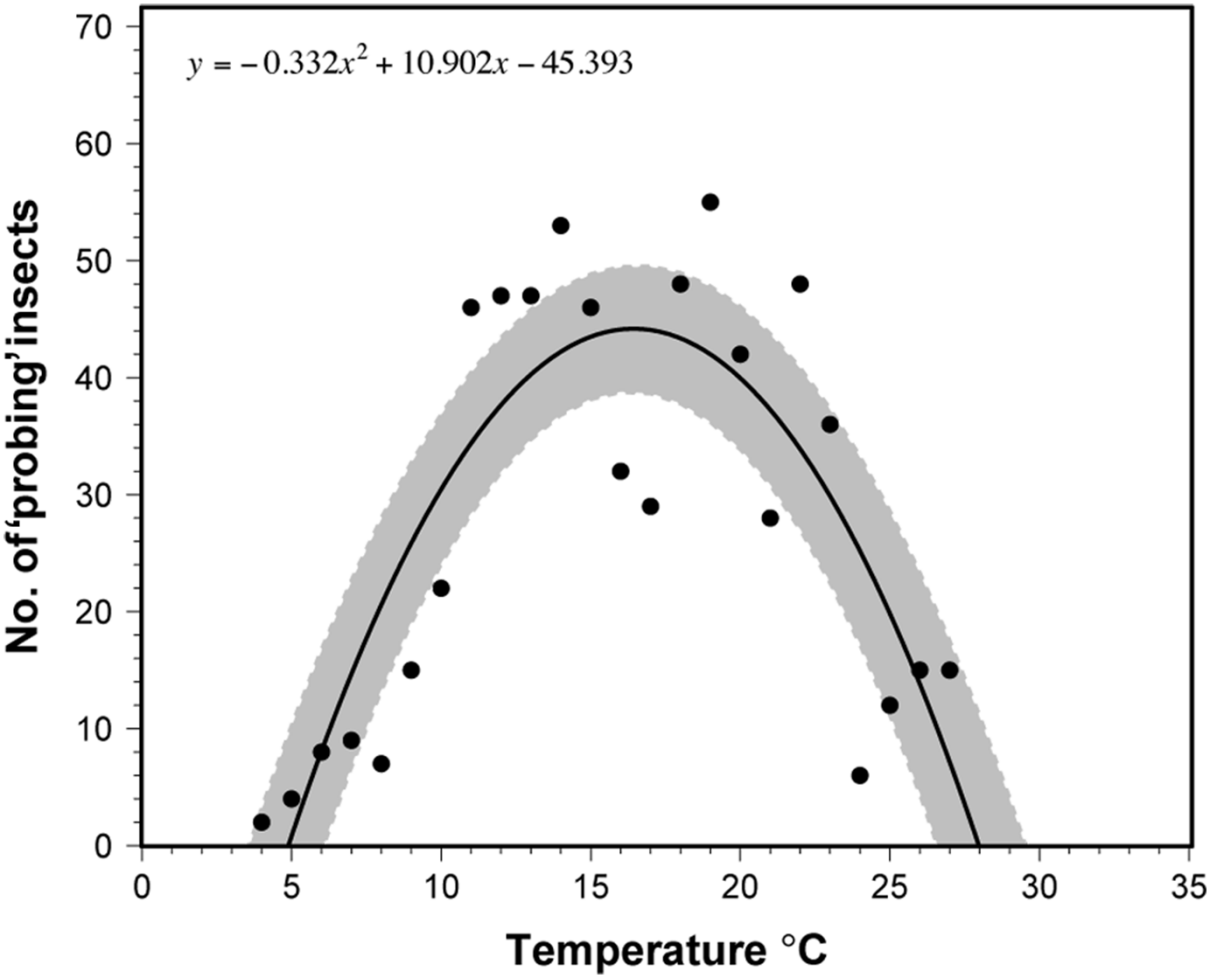
Temperature-dependent ‘probing’ activity curve of *H. halys*.

The number of insects that ‘probed’ at each of the temperatures tested was fitted with a second order polynomial model (*F* = 24.33; d.f. = 2,21; *P*<0.001, *r* ^2^ = 0.67; Figure 6). The lower and upper ‘probing’ thresholds predicted by the model were 4.89 and 27.97°C, respectively. A 95% confidence interval predicted that the minimal temperature at which ‘probing’ of *H. halys* could occur was at temperatures between 3.51 and 6.13°C, and that ‘probing’ would likewise cease at high temperatures between 26.53 and 29.60°C. The optimal ‘probing’ temperature was predicted to be approximately 16.5°C, and the 95% confidence interval predicted that the most conducive temperatures for feeding were between 16 and 17°C.

### Phenological effects on ‘probing’ activity

**Figure 7:**
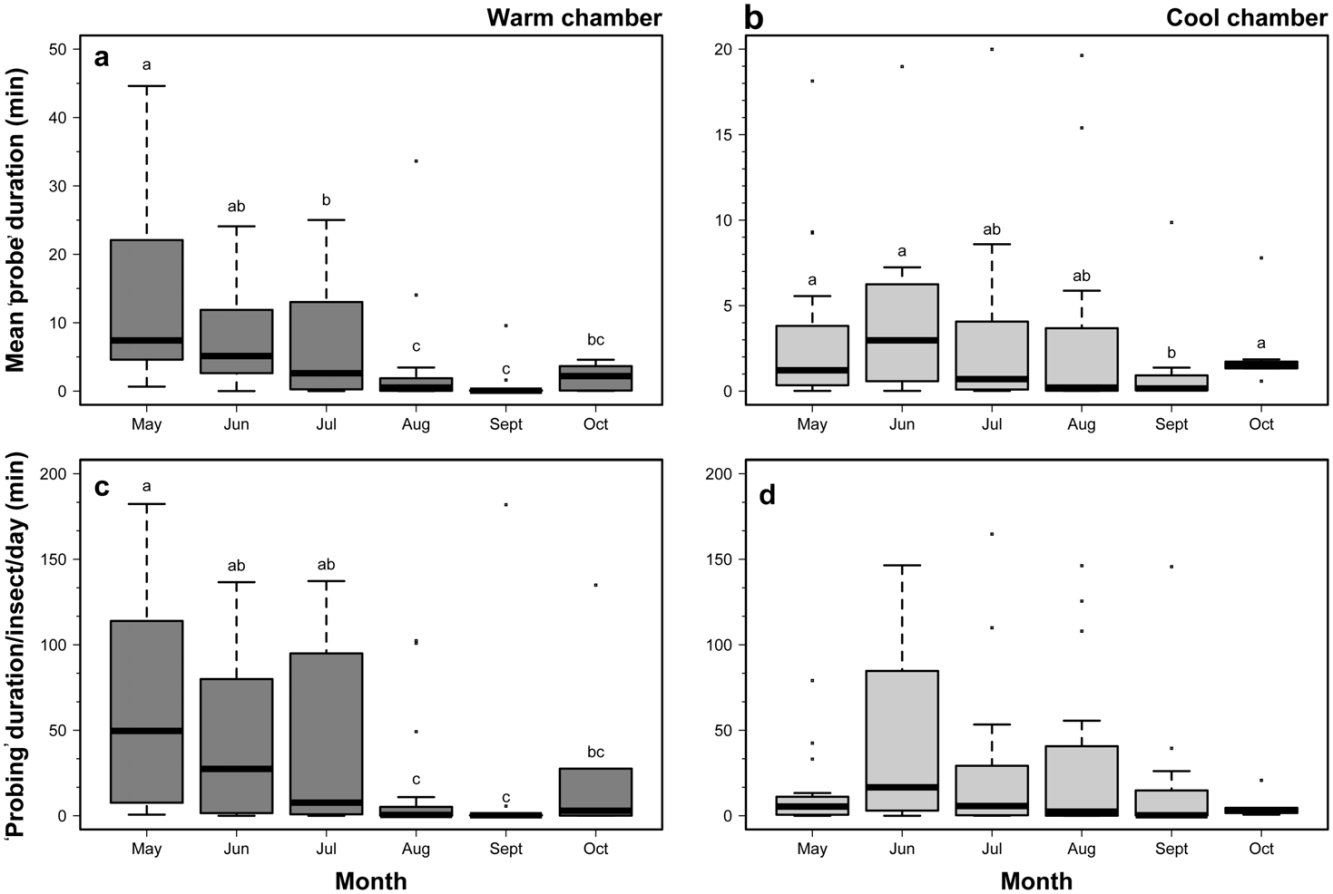
‘Probing’ duration and total ‘probing’ time for *H. halys* in each of warm and cool environmental chambers.

The duration of ‘probes’ depended on the month in which the assay was conducted in both the warm (*F* = 8.11; d.f. = 5,84; *P*<0.001; Figure 7a), and the cool environmental chambers (*F* = 2.38; d.f. = 5,101; *P* = 0.043; Figure 7b). Some of the longest mean ‘probe’ durations occurred in chambers programmed to represent early season conditions. However, there were fewer significant differences between mean durations for the individual months in the cool environmental chamber (LSD test; *P*<0.05; Figure 7b). In the warm chamber, the shortest mean ‘probe’ duration was in August and September compared with May, June, and July. October mean ‘probe’ duration doesn’t appear to fit trends suggesting it was a potential outlier. ‘Probe’ frequency was not correlated with the month of the bioassay in either environmental chamber (*P*>0.05). There were significant differences in total ‘probe’ duration per insect per day for months in the warm environmental chamber (*F* = 4.82; d.f. = 5, 84; *P*<0.001; Figure 7c), but not in the cool chamber (*F* = 1.30; d.f. = 5, 100; *P* = 0.272; Figure 7d). Similar to the ‘probe’ duration data discussed above, adult *H. halys* collected in May, June and July tended to display longer durations per day than in August and September (LSD test; *P*<0.05; Figure 7c).

### Diel periodicity

**Figure 8:**
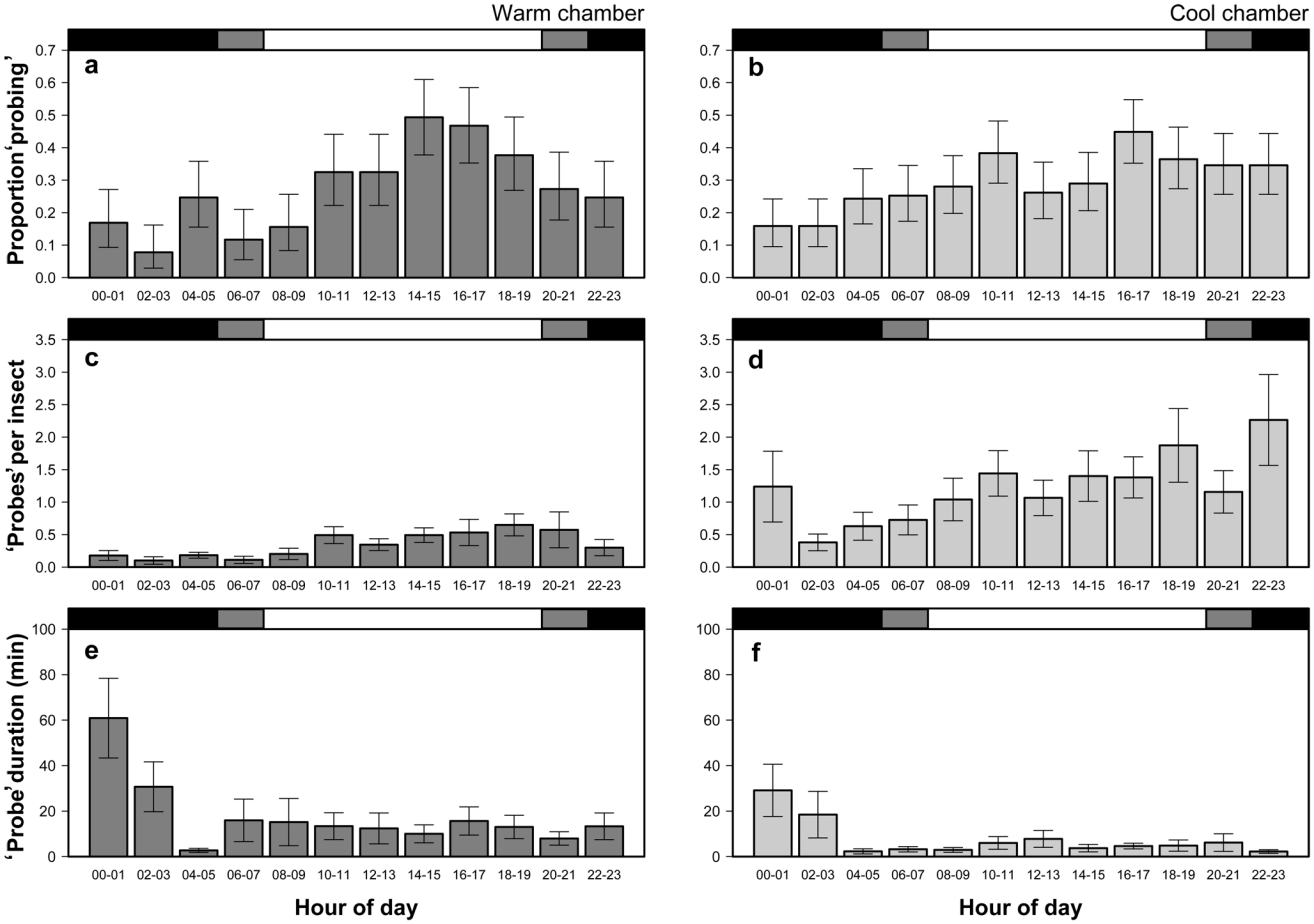
Diel periodicity for ‘probing’ behaviors (±95% CI); proportion of insects ‘probing’ (a–b), ‘probes’ per insect (c–d), and ‘probe’ duration of *H. halys* (e–f).

In the warm chamber, the proportion of ‘probing’ *H. halys* steadily increased from the start of photophase to the peak of 0.44 within the hours of 16∶00 and 17∶00 (Figure 8a). The number of ‘probes’ declined as scotophase approached, and the lowest proportion of adults engaged in ‘probing’ (0.12) occurred during scotophase between the hours of 02∶00 and 03∶00. In the warm chamber, the proportion of adults that ‘probed’ during photophase was 0.94, and was significantly higher than the proportion that ‘probed’ during scotophase (0.43; Figure 8a).

**Figure 9:**
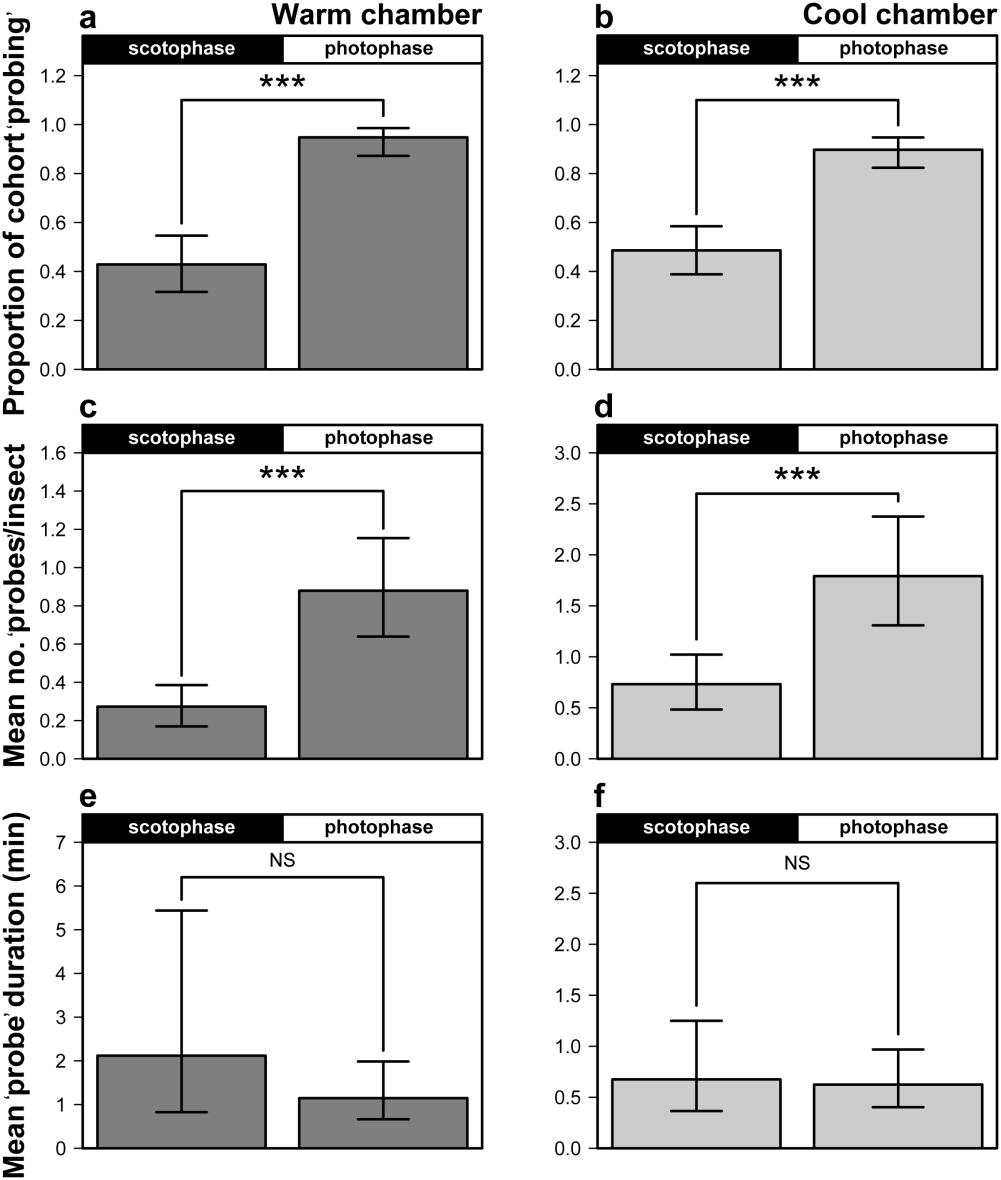
Diel ‘probing’ activity comparison (±95% CI); proportion of *H. halys* ‘probing’ (a–b); ‘probes’ per insect per day (c–d), mean ‘probe’ duration (e–f).

In the warm chamber, a significantly higher proportion of the insects ‘probed’ during photophase (0.95) compared to scotophase (0.43; binomial test; *P*<0.001; Figure 9a). Similarly, in the cold chamber, the peak proportion of ‘probing’ *H. halys* (0.36) was recorded within the hours of 18∶00 and 19∶00 (Figure 8b). The observed maximum proportion of *H. halys* ‘probing’ was 0.45, which occurred between the hours of 16∶00 and 17∶00. Nonetheless, the lowest predicted observed proportion of ‘probing’ insects (0.14) occurred during scotophase between the hours of 00∶00 and 01∶00. The proportion of insects ‘probing’ during photophase (0.89) was again significantly higher than the proportion feeding (0.49) during scotophase (binomial test; *P*<0.001; Figure 9b).

The mean number of ‘probes’ per insect that occurred within the 2 hr increments followed a similar pattern to the proportion of ‘probing’ insects in the warm and cool chambers. In the warm chamber, the frequency of ‘probing’ increased steadily after photophase began and reached a maximum (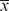 **= **0.58 ‘probes’ per insect) slightly later between 18∶00 and 19∶00. Again, ‘probing’ frequency decreased after scotophase began, reached a minimum (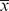 **= **0.11) during the hours 02∶00 and 03∶00. In the cool chamber, ‘probing’ events per insect increased steadily over the day to its peak (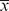 **= **2.26) between the scotophase hours of 22∶00 and 23∶00. The observed low (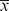 **= **0.38) occurred between the hours 02∶00 and 03∶00, but the predicted low (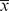 **= **0.78) occurred between 04∶00 and 05∶00. On average, *H. halys* probed more frequently during photophase compared with scotophase in both the warm (*t* = 4.78; d.f. = 327; *P*<0.001) and cool chambers (*t* = 3.84; d.f. = 376; *P*<0.001; Figure 9c–d).

In contrast to periodicity patterns discussed above, mean ‘probe’ duration peaked during scotophase in both warm and cool chambers (Figure 8e–f). In the warm chamber, the max observed mean (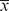 **= **60.90 min) occurred during scotophase in the hours 00∶00 and 01∶00, and then mean ‘probe’ duration declined and remained relatively flat during the photophase hours (Figure 8e). A very similar pattern was found in ‘probe’ duration data from the cool chamber, but the magnitudes of the means were numerically lower (Figure 8f). Despite the elevated mean ‘probe’ duration between the hours of 00∶00 and 04∶00, there were also some low mean ‘probe’ durations during scotophase; consequently, there wasn’t a statistical difference in mean ‘probe’ durations between scotophase and photophase for the data from the warm (*t* = 1.14; d.f. = 55; *P* = 0.259) or the cool (*t* = 0.21; d.f. = 103; *P* = 0.836) incubator (Figure 9e–f).

## Discussion

In this study we demonstrated the application of a simple EMIF system for assessing ‘probing’ activity, of large piercing-sucking hemipterans such as Pentatomidae. There have been several EMIF systems that exploit feeding through an electrified grid into a food item to complete an electrical circuit as a method to monitor feeding behavior of piercing-sucking insects [35], [47]–[49]. Our design is specialized for working with cut fruits and vegetables, and besides green beans, apple slices have been used with success (NGW, unpublished). This versatility will allow for additional studies on the influence of different food sources. Other potential use of this EMIF system is for bioassays to evaluate effects of feeding stimulants, deterrents, or insecticide treatments on ‘probing’ activity. The EMIF system is compact and can be used to monitor activity in small environmental chambers. Because the purpose of our system is to simply monitor presence or absence of proboscis contact with the food item, and not to capture high-resolution waveform data like EPG, the electromagnetic interference present in environmental control chambers is not a concern. Instead, our system allows laboratory studies to examine how temperature and photoperiod affect ‘probing’ behavior, and to efficiently replicate data for many insects. Another benefit of USB data acquisition devices is their channel capacity and relatively low cost compared to EPG systems. Although we utilized only 15 analog input channels in this study, there is potential to add many more channels by integrating more devices, or by using devices that have 80 or more analog input channels (*e.g.*, NI USB-6255; National Instruments Corporation). This high capacity for replication is feasible because our system requires minimal handling of insects and no operator attention during the trial.

Shearer and Jones (1996) provided the precedent for our current study. They used a horizontal orientation of the feeding cup in their study, but here we have modified the orientation of the cup to a vertical axis. As they noted, it is important that excretions produced by the insects fall away from the screen to avoid false positives. The vertical orientation of the feeding station cup in our system is based on a rearing protocol for *N. viridula* where food is placed over netting covering the tops of buckets containing stink bugs [55]. The vertical cup allowed *H. halys* a choice between feeding or basking at the top of the cup, or seeking refuge in the bottom of the cup. Observation of *H. halys* feeding *in natura* suggest that underside of leaves and fruits and vegetables are often utilized, and it is clear that this insect often feeds in an upside-down orientation. Use of articulated arms for the electrodes allowed us to adjust positions of individual beans at each station. Subtle variation in bean morphology necessitates that the electrodes can be positioned individually to increase surface area of the bean that is available to the insect. We also reduced the circuitry by utilizing a common +VDC source by charging one large copper screen per table instead of 8 individual screens. By having this common voltage source and all of the stations beneath, we were able to substantially reduce the size of the instrument.

When examining the data distributions for the duration of ‘probing’, as well as the number of ‘probing’ events, the insect is often briefly touching the food with its mouthparts, but clearly there is not enough time for penetration or salivation. This leads to distributions being heavily skewed because of many recorded ‘probes’ of short duration, often lasting only one second. It is likely that the short ‘probes’ are actually not probes by the strict definition that the food is actually penetrated by the stylets [41], but rather represent “labial dabbing” a behavior that is characterized by the exploration phase of the feeding behavior sequence of Auchenorrhyncha, Sternorrhyncha, and Heteroptera [56]. Labial dabbing can be described as repeated touching of the food with the labial tip, and may be accompanied by a drop of sheath saliva from the stylets. Hemipteran mouthparts are heavily endowed with external sensilla that take a hair or peg-like, or multi-lobe form [44], [57]. These structures play a role in the sensory aspects of feeding, and labial sensilla may be particularly important for assessing food source acceptability during this exploring phase.

Assuming acceptability of the food after the exploratory phase, test ‘probing’ (wherein the stylets are inserted) is likely to follow [56]. Test ‘probing’ may end abruptly or proceed into exploratory feeding and excretion of watery saliva for digestion and finally ingestion. These latter behaviors are expected to be longer in duration. Hemipterans are capable of salivating and ingesting multiple times without removing mouthparts by repositioning the tip of the stylets [58]. The salivary sheath is constructed from hardened protein-rich saliva that forms around the stylets, which may aid in sealing the wound against leakage, or lubricating or supporting stylets [8], [56], [44], [59], [60]. Counting of salivary deposits on the surface of a food item is often used as a method to gauge feeding activity of stink bugs [34], [61], [62]. However, it is difficult to distinguish whether the stylets actually penetrated the substrate or if ingestion took place from the presence of sheath saliva. This is because sheath saliva hardens as it oxidizes [56], and can transfer to the food during the exploratory phase of feeding, leaving what appear to be salivary sheaths that are not accompanied by a puncture [63].

While it is clear that a fair amount of labial dabbing and test ‘probing’ would occur in nature prior to salivation and ingestion, there is also potential that our experimental conditions led to increased ‘probes’ of short duration. One condition that may have led to an increased number of short stylet contacts was the use of field-collected insects. These insects were collected from a variety of hosts but never from green beans. Thus, not only were they unfamiliar with this standard stink bug laboratory food, but they were collected at various levels of satiation, nutritional status, and age. A second potential condition leading to short ‘probes’ was that the insects may be challenged in negotiating the screen to find a suitable feeding position. A third condition that may have led to short ‘probes’ is the execution of these experiments under variable and dynamic environmental conditions, including temperatures that were not conducive to feeding. This is supported by the result that cooler temperatures caused shorter duration ‘probes’. It is also important to recognize that the high number of short ‘probes’ may also be related to suboptimal ratio of voltage to input impedance. It is known that very small currents can have large behavioral implications in EPG [64].

The longer extremes in the mean ‘probe’ duration recorded for *H. halys* (up to 184 minutes) probably reflect establishment of salivary sheaths and perhaps multiple salivary deposits. Depieri and Panizzi (2011) examined ‘probing’ duration in four pentatomid species and found that mean ‘probe’ duration ranged from 71–133 min. However, their experiments were performed on starved laboratory insects under warm temperatures (25°C) and only ‘probing’ with vertical head movements was recorded observationally. This suggests that exploratory labial dabbing was not a behavior that captured in that study. This may help explains why our mean ‘probe’ durations were lower. The few other studies on ‘probe’ duration of pentatomids have noted mean ‘probing’ durations lasting from 70–90 minutes, although these experiments are typically based on periodic observational data and again, do not include labial dabbing behavior [34], [65]. A problem with periodic visual observational data is observing the brief dabbing events, and the difficulty in distinguishing discrete ‘probes’. Clearly, these problems can lead to overestimation of probe durations. The amount of damage on soybean from stink bug has been correlated with feeding time [31], presumably because longer feeding correlates with salivation and digestion of plant material. Future studies should characterize and link EMIF activity data with crop damage and formation or lack of salivary sheaths, as this could help with diagnosis of damage resulting from *H. halys.* Behavioral studies, waveform characterization from EPG data, and histological studies on the food item will be necessary to quantify and distinguish different feeding behaviors of *H. halys.* Diverse feeding strategies including stylet sheath feeding, cell-rupture feeding or “lacerate-and-flush” are used by pentatomids depending on the type of plant tissue that is targeted [5], [43], [44], [56]. The type of basic EMIF data used in this study has very limited capacity to distinguish specific feeding strategies used by *H. halys*, and EPG will have an important role to play for determining specific feeding strategies of this pest.

The effect of environment on feeding behavior has not been widely examined in in EPG or EMIF studies, partly because it is difficult to adapt EPG systems to controlled environment chambers as experiments must be conducted in a Faraday cage. However, at least one study has performed EPG outdoors [60]. Similar to that study, we found temperature had important effects on *H. halys* ‘probing’ behavior. In the cooler environmental chamber, *H. halys* probed more frequently, representing more labial dabbing than the insects that were kept in the warmer environmental chamber. Because they were so active, the disparities observed in mean ‘probing’ durations may be linked to a temperature-dependent physiological component of feeding, rather than a metabolic limitation from temperature. The higher frequency of ‘probing’ and the shorter mean ‘probe’ duration balanced out under cool temperatures and resulted in similar total ‘probing’ time per day between the two temperature treatments. However, the current study cannot address whether ‘probing’ duration reflects nutritional value for *H. halys*. Hemipteran watery saliva, which is secreted into the feeding site, contains a cocktail of digestive enzymes, including amylase, proteinases, and pectinase, which dissolve cell walls and contents [8], [56], [66]–[68]. The resulting slurry is then imbibed by the insect, and digestion continues in the gut. However, enzymatic reactions are temperature dependent, and are more efficient at warmer temperatures [69], [70]. Thus, the amount of nutrients acquired could be less for the cooler insects, and potentially left the insects unsatisfied. This might help explain why our insects probed more frequently in the cooler chamber.

It is clear from our study that temperature was a key variable in explaining when individual stinkbugs tended to actively ‘probe’. Interestingly, the lower threshold for ‘probing’ activity is far below the minimum temperature threshold for development, which is located between 12 and 15°C [71]–[73]. Thus, ‘probing’ by *H. halys* may occur even when development may not. Although developmental temperature thresholds apply primarily to molting in immature insects, in adult insects they may relate to sexual maturation or other processes. Our data predict that the optimal ‘probing’ temperature range is just above the minimum temperature threshold for development. The predicted upper temperature threshold for ‘probing’ may appear surprisingly low, as *H. halys* can be observed feeding on very hot days. However, it must be noted that temperatures experienced by *H. halys* feeding within a canopy can potentially be much cooler than air temperatures due to interaction of factors such as evapotranspiration, radiation absorption, and other processes [74]. Furthermore, *H. halys* is not an extraordinarily heat-tolerant insect. The upper threshold for development of *H. halys* has been determined to be 35.76 and 36.5°C [71], [72], which is at or above the upper ‘probing’ threshold predicted by our data.

There were phenological differences in feeding activity between the different months when the assays were conducted. These differences may have reflected different environmental conditions, and they also coincide with what is known about the phenology of *H. halys* in temperate regions of the northern hemisphere [71], [75], [76]. In the spring months, *H. halys* adults emerge from overwintering sites where they have aggregated during winter. They then move to host plants and feed for the first time in months before beginning sexual maturation. Coinciding with this period are some of the longest photoperiods and warmest temperatures of the season. The combination of nutritional needs, warm temperatures, and a high degree of phenological synchrony among *H. halys* at this time could be the reason that there was higher feeding activity in May and June than in other months. In Oregon, there is potential for two generations of *H. halys* per year (Shearer and Wiman, unpublished), and after mid-summer the population collected in beat samples collected from host plants is comprised of a mix of residual overwintered adults, summer adults, and nymphs. In the fall, the stink bug largely cease feeding, and begin to move to winter aggregation sites as they enter diapause. Our data seemed to reflect this phenology, as the lowest adult feeding activity was found in August and September when winter aggregation behavior is occurring. Future studies will examine feeding by nymphs during the latter summer months, as they may potentially be a major source for late-season crop damage.

Using an EMIF system similar to the present study, Shearer and Jones (1996) found that *N. viridula* had the longest ‘probing’ periods during scotophase [35]. Similarly, Krupke et al. (2006) observed that the pentatomid *Euschistus conspersus* Uhler was more frequently observed feeding during scotophase [77]. While we did find that a substantial amount of feeding activity of *H. halys* occurred during scotophase, including some of the longest mean ‘probe’ durations, this activity was lower or no different from the ‘probing’ activity levels during photophase over all. Our data suggest that feeding periodicity of Pentatomidae could be more dependent on temperature than on photoperiod. Shearer and Jones (1996) conducted their study using laboratory insects under constant temperature (21°C), while the Krupke et al. study had natural photoperiod and temperature with field-collected insects in the greenhouse, but observations were only conducted during warm months (late August and early September). The Willamette Valley OR, has relatively cool nights and such fluctuations of temperatures were reflected by our controlled temperature cabinet simulations as it occurs prior to dawn (photophase). We believe that field collected-insects were therefore preconditioned to very similar daily temperature fluctuations. Mean ‘probe’ duration during scotophase declined in our study as temperatures dropped in approach to the daily low. On the few summer nights where temperatures do not drop in the Willamette Valley, and in warmer climates, ‘probing’ duration would likely remain high throughout scotophase. However, the relative importance of temperature and photoperiod as drivers of feeding behavior needs additional investigation.

There are several factors that determine crop damage from stink bugs including density of the bugs and feeding intensity. Our study focused only on the latter and we found that ‘probing’ activity of *H. halys* adults was highest in the early to mid-season. This is a time when many crops affected by *H. halys* are still developing, and fruit crops may be particularly susceptible to damage such as cat facing that becomes apparent at harvest. Some of the most severe crop damage usually begins to occur in the late season when stink bug densities are relatively higher and there is mass immigration into crops [20], [78]. However, ‘probing’ activity of adult *H. halys* is predicted to be lower at these times, and thus the insect is less damaging on a per-capita basis. Pentatomid nymphs may have increased feeding activity compared with adults [34] and nymphs are abundant in the late season. Thus, it will be important in future studies to evaluate feeding behavior of *H. halys* nymphs in order to determine their relative potential to cause damage.
